# The efficacy of branched-chain amino acid granules to restore phagocytic activity in cirrhosis patients, a randomized controlled trial

**DOI:** 10.3389/fnut.2023.1142206

**Published:** 2023-05-12

**Authors:** Naichaya Chamroonkul, Natthapat Rujeerapaiboon, Pimsiri Sripongpun, Apichat Kaewdech, Teerha Piratvisuth

**Affiliations:** ^1^Gastroenterology and Hepatology Unit, Division of Internal Medicine, Faculty of Medicine, Prince of Songkla University, Songkhla, Thailand; ^2^Gastroenterology Endoscopy and Motility Center, Ramathibodi Hospital, Mahidol University, Ratchathewi, Thailand; ^3^NKC Institute of Gastroenterology and Hepatology, Songklanagarind Hospital, Prince of Songkla University, Songkhla, Thailand

**Keywords:** cirrhosis, branch-chained amino acid, innate immunity, phagocytosis, cirrhosis associated immune dysfunction

## Abstract

**Background:**

Infection is a detrimental complication among cirrhotic patients, leading to major morbidity and mortality. Reduction in phagocytic activation, as part of immunoparesis, is a distinctive key component of cirrhosis-associated immune dysfunction (CAID) and predicts the development of infection. However, there are limited data on immunotherapeutic approaches to restore phagocytosis.

**Aims:**

We aimed to determine the effect of branched-chain amino acid (BCAA) granules on phagocytic activity in patients with CAID.

**Methods:**

In this double-blind randomized controlled trial, Participants were randomly assigned (1:1 ratio stratified by Child-Pugh status) to receive either BCAA granules or placebo. In the 3rd and 6th months, phagocytic activity was assessed by flow cytometry. The primary endpoint was the restoration of innate immunity at the 6th month, defined as ≥75% phagocytic activity; the secondary endpoints were the accretion of phagocytic activity and hospitalization due to infection.

**Results:**

A total of 37 patients were included. There were no differences among the patients in the baseline characteristics and phagocytic activity. At the 6th month, a higher proportion of patients with phagocytic restoration was observed in the BCAA granule group compared to the placebo group (68 vs. 5.6%, *p* < 0.001). The mean phagocytic activity was 75.4 and 63.4% in the BCAA granule and placebo groups, respectively (*p* < 0.001). Progressive accretion of phagocytic activity was observed during the 3rd and 6th months. There was no difference in hospitalization due to infection (3 vs. 2 events, *p* = 0.487).

**Conclusion:**

Our results suggest that BCAA granules significantly restore phagocytic activity across various stages of cirrhosis. A longer follow-up period is required to demonstrate infection prevention.

**Clinical Trial Registration**: www.clinicaltrials.in.th, TCTR20190830005.

## Introduction

Cirrhosis-associated immune dysfunction (CAID) has been coined as a complication of cirrhosis (1–3). Recent studies demonstrated that the proportion of individuals with infectious complications among patients with CAID is 34% per year at any stage of liver cirrhosis compared to 5% in the healthy population (2, 4). Moreover, CAID can lead to further liver decompensation and result in mortality (mortality rate: up to 63% in 1 year) (5, 6). Thus, CAID is predictive of infection-related casualties, organ dysfunction, and 90-day mortality (7–12).

Cirrhosis-associated immune dysfunction comprises two syndromic spectrums of immune alterations, including acquired immunodeficiency and systemic inflammation; this is a consequence of chronic immune system stimulation and homeostasis perturbation (13, 14). These syndromic spectrums indicate immune dysfunction status leading to infectious susceptibility and further liver decompensation. Several studies have demonstrated that the greater progression of liver cirrhosis, the greater severity of reduction in immune capacity (11). This is evidenced by the exhaustion of the circulating innate immune response-related cells and decrease in phagocytic activity despite the presence of infection.

The innate immune system plays a crucial role in protecting hosts against microorganisms via various mechanisms. Innate immune activation requires priming by microorganisms, pathogen-associated molecular patterns (PAMPs), and damage-associated molecular patterns (DAMPs), orchestrating with the metabolic regulation by the mammalian target of rapamycin (mTOR). Recent studies have proposed several mechanisms that cause abnormalities in the immune cells in cirrhosis (4, 13, 15). These abnormalities compromise the effector function of the immune cells and result in immunodeficiency. The underlying mechanisms include loss of hepatic immune cells, hepatic fibrosis, immune cell exhaustion due to the protracted activation of toll-like receptors (TLRs), and metabolic abnormalities (13).

Branched-chain amino acids (BCAAs; leucine, isoleucine, and valine) are essential amino acids. A large-scale study in Japan demonstrated the benefit of oral BCAA supplementation in reducing liver complications, such as liver failure, esophageal varices rupture, hepatocellular carcinoma, and death (16). Furthermore, BCAAs are integrated by immune cells as part of the cell proliferation process. They activate the mTOR pathway, a central signaling pathway of the immune microenvironment (17, 18). Additionally, a translational study demonstrated that BCAAs reduce bacterial translocation, lipopolysaccharide-binding protein (LBP) expression, and TLR-4 activation; these are essential mechanisms leading to immune exhaustion (19). A recent pilot study of 10 decompensated cirrhotic patients showed that BCAAs could significantly improve neutrophil phagocytic capacity (20).

There are limited immunotherapeutic approaches to modulate the immune response in CAID. In this regard, BCCA granules have been shown to have considerable benefits and satisfactory safety profiles in cirrhotic patients. Therefore, our study aimed to determine the effect of BCAA granules on the innate immunity of patients with cirrhosis.

## Materials and methods

### Study design

We conducted a prospective, randomized, double-blind, placebo-controlled study that recruited cirrhotic patients at Songklanagarind Hospital, a tertiary care university hospital in Thailand, between February 2020 and February 2021. The trial protocol was approved by the Institutional Review Board of the Faculty of Medicine, Prince of Songkla University (REC 62-275-14-1). The study was conducted according to the principles of the Declaration of Helsinki. This study has been registered at www.clinicaltrials.in.th (TCTR20190830005). Informed consent was obtained from all the participants.

### Participants

Patients diagnosed with cirrhosis either by imaging or histopathology, aged between 18 and 70 years and with less than 75% phagocytic activity were included in the trial. Patients meeting any of the following criteria were excluded: previous hospitalization due to infection within 3 months, active malignancies, life expectancy <9 months, alcohol cessation <6 months, concurrent treatment with steroids or any immunosuppressive agents, uncontrolled diabetes mellitus (defined by HbA1c ≥8%), human immunodeficiency virus infection, estimated glomerular filtration rate (eGFR) <30 mL/min/1.73 m^2^, taking BCAAs within 3 months before enrolment, pregnancy or lactation, and Model For End-Stage Liver Disease (MELD) score ≥ 20.

### Outcomes

The primary outcome was the percentage of patients with phagocytic function restoration, defined as ≥75% phagocytic activity (comparable to that in a healthy population) (12, 18). The key secondary efficacy outcomes included phagocytic accretion, hospitalization due to infection, and changes in inflammatory biomarkers.

### Clinical and laboratory parameters

Routine laboratory investigations, including liver biochemistry tests, complete blood count, coagulogram, serum creatinine, and serum sodium, were performed at enrolment and at the 3rd and 6th month of the study. In addition to routine laboratory tests, biomarkers of systemic inflammation (SI), including erythrocyte sedimentation rate (ESR), C-reactive protein (CRP), and serum ferritin, were assessed at an ISO-certified laboratory on the same day as the phagocytic activity measurement at months 0, 3, and 6.

### Phagocytic activity measurement

Phagocytosis was assessed by a well-trained biochemist at the Department of Biomedical Sciences and Biomedical Engineering, Faculty of Medicine, Prince of Songkla University. To evaluate phagocytosis, we used the pHrodo™ Red *E. coli* BioParticles® Phagocytosis Kit for flow cytometry (Thermo Fisher Scientific Corporation, California, United States). This assay has been validated and used in previous studies (21–23).

#### Preparation of BioParticles®

Lyophilized pHrodo™ Red *E. coli* BioParticles® was reconstituted in an uptake buffer at 1 mg/mL (pH 7.4). The vials were vortexed for 60 s, sonicated for 5 min, and placed on ice for an additional 10 min before use.

#### Analysis of phagocytic activity

Peripheral whole blood samples from the participants were collected into heparinized tubes and stored at 4°C. The analysis was performed within 4 h of blood sampling. Once the analysis was performed, the whole blood and prepared BioParticles® were placed on ice for 10 min before being used. Two sets of negative control tubes (NC) and positive control tubes (PC) were incubated, one set each at 4 and 37°C (total four tubes). The BioParticles® (20 μL) were added to the PC tubes containing 100 μL of the sample solution. One set of NC and PC tubes was placed on ice, and the other set was placed in a 37°C water bath and incubated for 15 min. Red blood cells were lysed at room temperature for 5 min using 100 μL of RBC lysis buffer. The samples were then centrifuged at 350 × *g* at room temperature for 5 min, washed twice with staining buffer B and analyzed by flow cytometry.

#### Flow cytometric analysis

**Figure 1:**
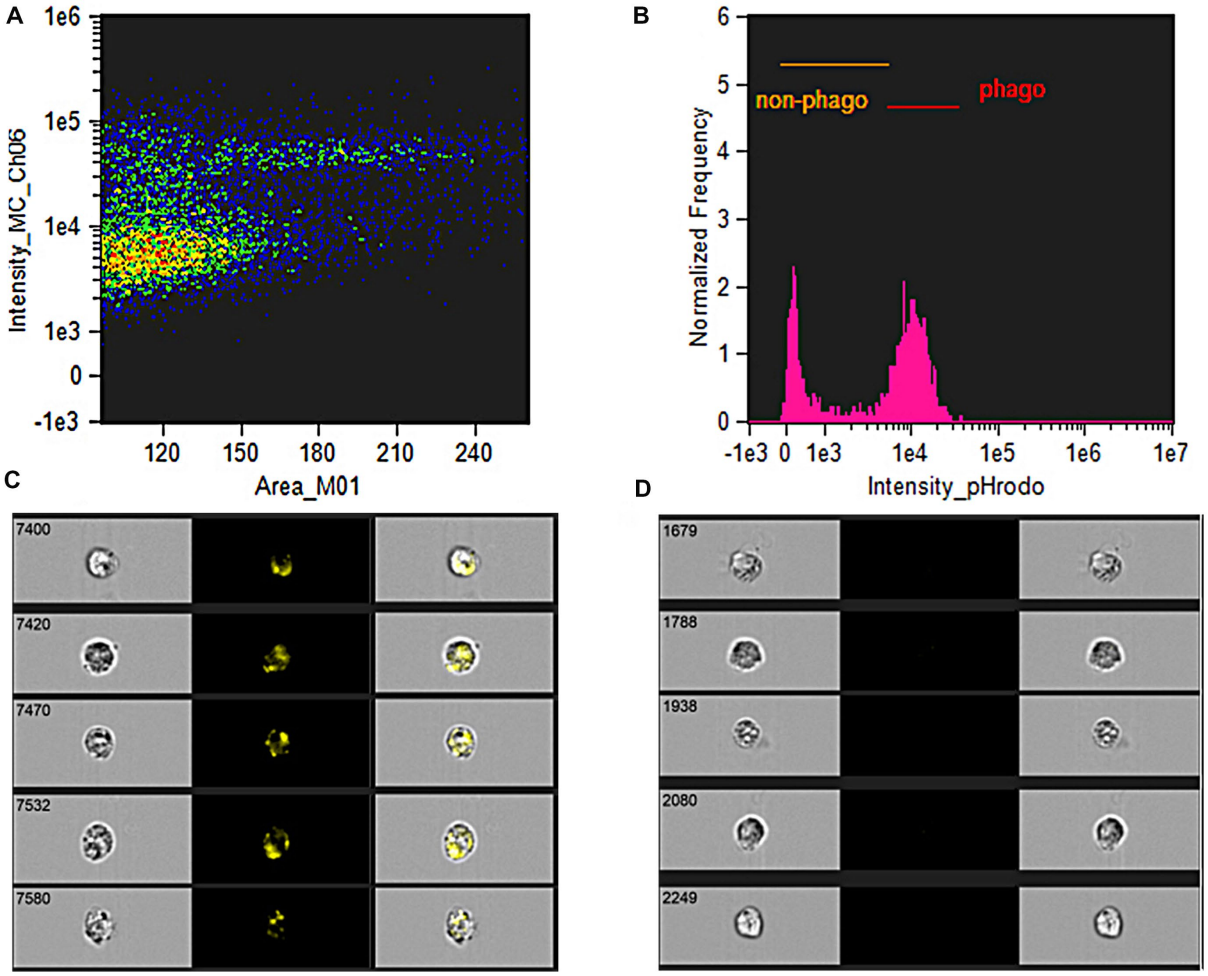
Phagocytic activity analysis. **(A)** Side-scattered plot demonstrated the cells selected for analysis, which granulocytes were further gated by the imaging-guided flow cytometry. The demonstrated colors of green, yellow, orange, and red represented the density of cells from low to high density in a particular area. **(B)** Proportion of non-phagocytic and phagocytic granulocytes was analyzed by fluorescence intensity after gating specified granulocytes. **(C)** The image flow cytometry demonstrated the high-yellowish intensity of fluorescence-tagged *Escherichia coli* were engulfed into the intracellular representing phagocytic granulocytes. **(D)** The image flow cytometry demonstrated the non-yellowish intensity of fluorescence representing non-phagocytic granulocytes.

Flow cytometry (ImageStream®^x^Mk II Imaging Flow Cytometer, Luminex, United States) was performed to detect the percentage of phagocytosis or functioning granulocytes (Figures 1A–D). Functioning granulocytes were detected using flow cytometry with a 488 nm argon-ion laser excitation filter and a 585/25 nm emission filter. A detailed analysis of the percentage and intensity of phagocytosis was performed using the IDEAS® 6.2 software.

### BCAA granules

Each packet of BCAA granules (4.15 g; LIVACT®, Ajinomoto Pharmaceuticals Co., Ltd., Japan; registration number: 2C 44/59, ATC number: A16AA000101) contained the following ingredients: L-Isoleucine 952 mg, L-Leucine 1,904 mg, and L-Valine 1,144 mg. Patients in the BCAA granule (intervention) group were instructed to consume the contents of one sachet each after breakfast, lunch, and late evening (a total of three sachets per day) for 6 months. The placebo (control) group received the placebo containing isocaloric starch without BCAA supplementation. The placebo sachet was identical to the intervention sachet, and the control group received the exact instructions as the intervention group. The researchers provided a study diary to the participants. The participants were instructed to make diary entries each time they took the study agent; they were asked to bring the emptied sachets back to the investigators at every visit during the follow-up period. Adherence to the investigational drug was considered adequate when ≥80% of the prescription was taken by the patient. Furthermore, the patients were asked to complete a 7-day food recall record before each follow-up period.

### Data collection and sample size calculation

The eligible participants were randomized and stratified by the Child-Pugh status in a block of four using a computer generation, which was placed in sealed envelopes. The institute’s research assistant was subsequently assigned to unseal the numbered containers and allocated the investigation products to participants. The physicians and the biochemist were blinded since the identification of blood samples and phagocytic results were concealed.

A previous single-arm study in Japan reported a proportion of phagocytic restoration rate was 100% after 3 months (mean phagocytic activity increased from 65 to 79%), with time-dependent improvement (21). Accordingly, assuming a 10% dropout rate, we expected a proportion of phagocytic restoration at 70% at the 6th month of the study with an absolute difference of 20%. Overall, we determined that a total of 33 patients would be required to have 90% power at a two-sided significance level of 5%.

### Statistical analysis

Intention-to-treat (ITT) analysis was performed to assess the primary and secondary efficacy endpoints. The last observation carried forward method was used to impute missing values. Changes in phagocytic percentage were compared using the *t*-test and Chi-squared test. Differences in liver biochemistry and inflammatory markers, including ESR, CRP, and ferritin, were assessed using the Wilcoxon rank-sum test and *t*-test, as appropriate. All statistical testing was two-sided and was performed at a 5% level of significance. Statistical analyses were conducted using the R software version 4.0.3 (Vienna, Austria, 2020).

## Results

### Study population

**Figure 2:**
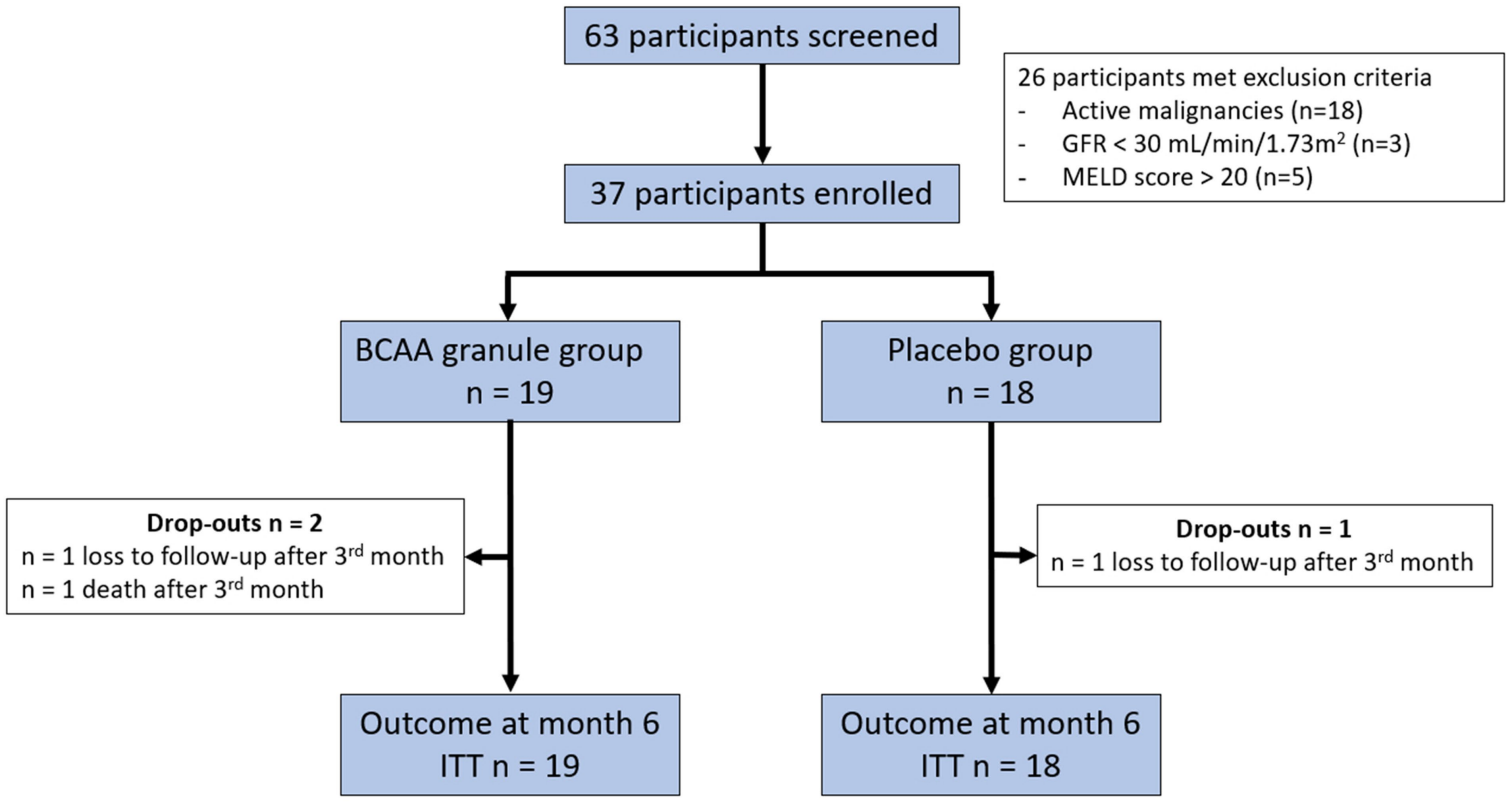
CONSORT flow chart of patients of the 63 participants screened, 37 fulfilled both the inclusion and exclusion criteria. The included participants were randomized and analyzed according to intention-to-treat (ITT) analysis. The participants were followed up for 6 months. BCAA, branched-chain amino acid; GFR, glomerular filtration rate; MELD, Model for End-Stage Liver Disease; and ITT, intention-to-treat.

A total of 63 patients were screened between February 2020 and February 2021. Of these, 37 participants were enrolled in the study: 19 patients in the BCAA granules group, 18 patients in the placebo group (Figure 2). Overall, 34 (91.9%) patients completed the study, and 3 (8.1%) patients discontinued early. Two patients in each group died after the 3rd month of the study. One patient in the BCAA granule group was lost to follow-up. The demographic and baseline characteristics of the patients were similar between the two groups (Table 1). The median age was 65 (IQR: 60.5–69) years in the BCAA granule group and 62.54 (IQR: 58.2–67.5) years in the placebo group. The majority of the enrolled patients were women (47.4% in the BCAA granule group, 77.8% in the placebo group), and 70.3% were Child-Turcotte-Pugh (CTP)-A. The main etiologies of cirrhosis in the participants included viral hepatitis (*n* = 18, 48.6%), alcohol consumption (*n* = 10, 27%), non-alcoholic fatty liver disease (NAFLD; *n* = 5, 13.5%), and other causes (*n* = 4, 10.8%); the etiology distribution did not differ between the two groups. There were no significant differences in liver biochemistry parameters, including total bilirubin (TB), direct bilirubin (DB), albumin, international normalized ratio (INR), as well as inflammatory markers and MELD-Na between the BCAA granule and placebo groups.

**Table 1:** Baseline characteristics of the enrolled participants.

Variable	BCAA granule group (*n* = 19)	Placebo group (*n* = 18)	*p* value
Age, years, median (IQR)	65 (60.5–69)	62.5 (58.2–67.5)	0.337
Female sex, *n* (%)	9 (47.4)	14 (77.8)	0.117
Etiology, *n* (%)			0.86
Viral hepatitis	8 (42.2)	10 (55.6)	
Alcohol	6 (31.6)	4 (22.2)	
NAFLD	2 (10.5)	3 (16.7)	
Others^†^	3 (15.8)	1 (5.6)	
LFT			
TB (mg/dL), median (IQR)	1 (0.8–2.5)	0.9 (0.6–2.1)	0.403
DB (mg/dL), median (IQR)	0.6 (0.4–1.2)	0.5 (0.3–1)	0.738
AST (U/L), median (IQR)	47 (24.5–42)	43.5 (35.5–54.8)	0.412
ALT (U/L), median (IQR)	31 (24.5–42)	37.5 (28.8–43.8)	0.429
ALP (U/L), median (IQR)	108 (95–177)	111 (93.8–151.8)	0.891
Albumin (g/dL), mean (SD)	3.5 (0.7)	3.7 (0.6)	0.375
Globulin (g/dL), median (IQR)	3.8 (3.4–4.8)	3.8 (3.6–4.1)	0.647
CTP, *n* (%)		*p* = 0.292	0.406
CTP-A	12 (63)	14 (78)	
CTP-B	5 (26)	3 (17)	
CTP-C	2 (11)	1 (5)	
WBC (x10^3^/μL), mean (SD)	5.3 (1.6)	5.2 (2.4)	0.838
ANC (x10^3^/μL), mean (SD)	3 (1)	3 (1.7)	0.99
INR, mean (SD)	1.3 (0.2)	1.3 (0.2)	0.608
Cr (mg/dL), mean (SD)	0.9 (0.2)	0.8 (0.3)	0.574
Na (mmol/L), median (IQR)	137.6 (135.9–139.6)	138.1 (137.4–139.2)	0.513
MELD Na, mean (SD)	12.3 (4.5)	11.9 (2.8)	0.733
ESR (mm/h), mean (SD)	39.7 (29)	38.3 (23)	0.867
CRP (mg/L), median (IQR)	1.3 (0.8–3.2)	2.7 (0.9–4.3)	0.354
Ferritin (ng/mL), median (IQR)	234 (126.5–277)	167 (83.4–293.2)	0.457
Phagocytic activity (%), mean (SD)	61.4 (6.7)	61.1 (6.4)	0.878

BCAA, branched chain amino acid; LFT, liver function test; ALT, alanine aminotransferase; ANC, absolute neutrophil count; AST, aspartate aminotransferase; Cr, creatinine; CTP, Child-Turcotte-Pugh; DB, direct bilirubin; INR, international normalized ratio; MELD, Model for End-stage Liver Disease; Na, sodium; NAFLD, non-alcoholic fatty liver disease; TB, total bilirubin; WBC, white blood cell; CRP, C-reactive protein; CTP, Child-Turcotte-Pugh score; and ESR, erythrocyte sedimentation rate.

† Other causes of cirrhosis included cryptogenic and primary biliary cholangitis.

The mean baseline phagocytic activity was 61.4 ± 6.7% in the BCAA granule group and 61.1 ± 6.4% in the placebo group.

### Efficacy endpoints

**Figure 3:**
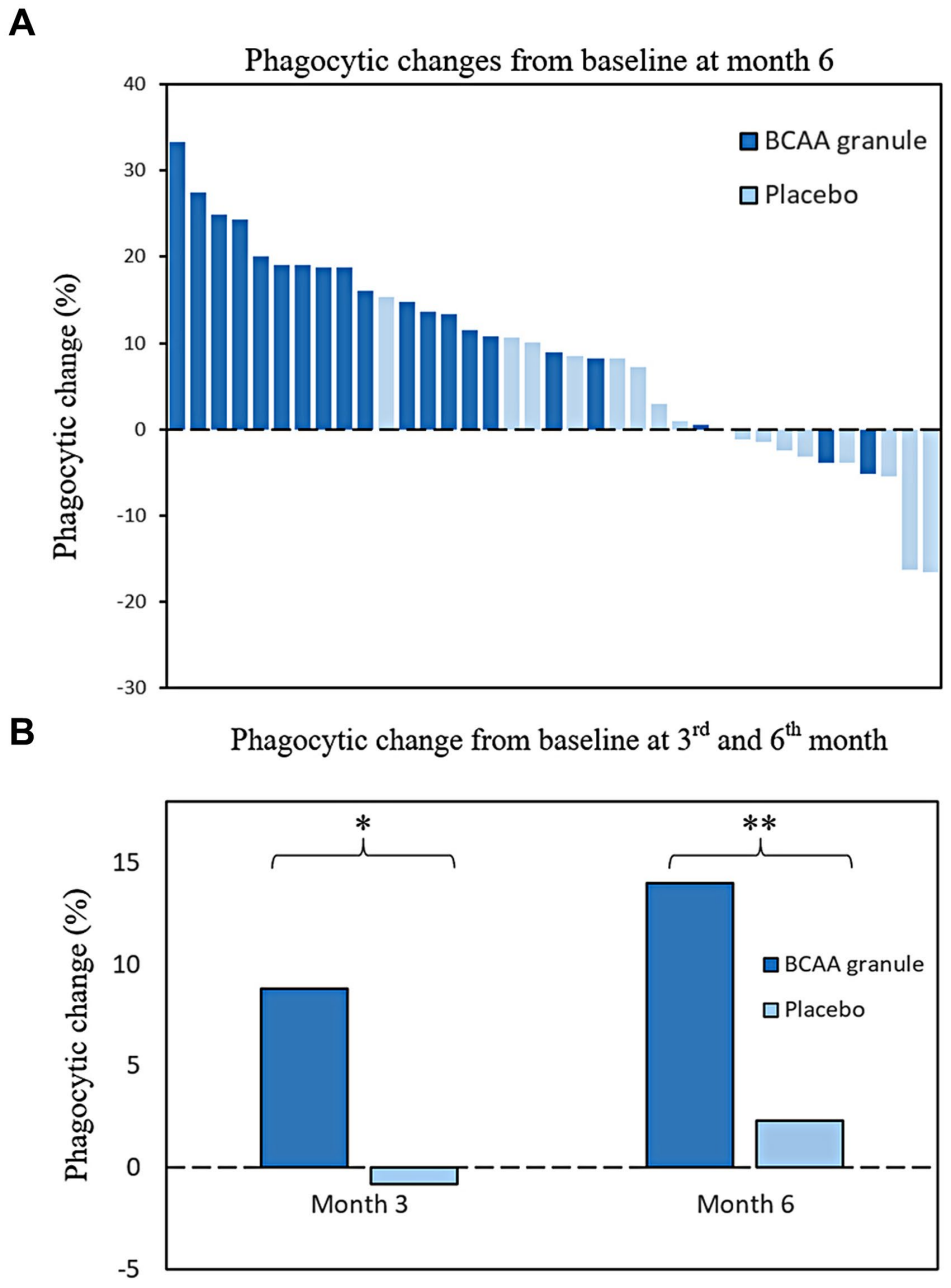
Changes in phagocytic activity from baseline at months 3 and 6 **(A)** Distribution of patients with diverse changes in phagocytic responses from baseline at month 6; **(B)** Significant changes in mean phagocytic activity were observed in the BCAA granule group compared to the placebo group at month 3 (8.8 ± 10.9% vs. −0.8 ± 7.9%, ***p = 0.004). The result was in accordance with month 6; a higher improvement in the mean phagocytic activity from baseline was observed in the BCCA granule group than in the placebo group (14 ± 10.7 vs. 2.3 ± 9.6%, *p = 0.001). BCAA, branched-chain amino acid.

After supplementation with either BCAA granules or placebo, a diverse trend in phagocytic responses was observed in the two groups (Figure 3A). A significantly higher mean phagocytic accretion from baseline was observed in the BCCA granule group compared to that in the placebo group at month 3 (8.8 ± 10.9 vs. −0.8 ± 7.9%, *p* = 0.004) and month 6 (14 ± 10.7 vs. 2.3 ± 9.6%, *p* = 0.001; Figure 3B).

**Figure 4:**
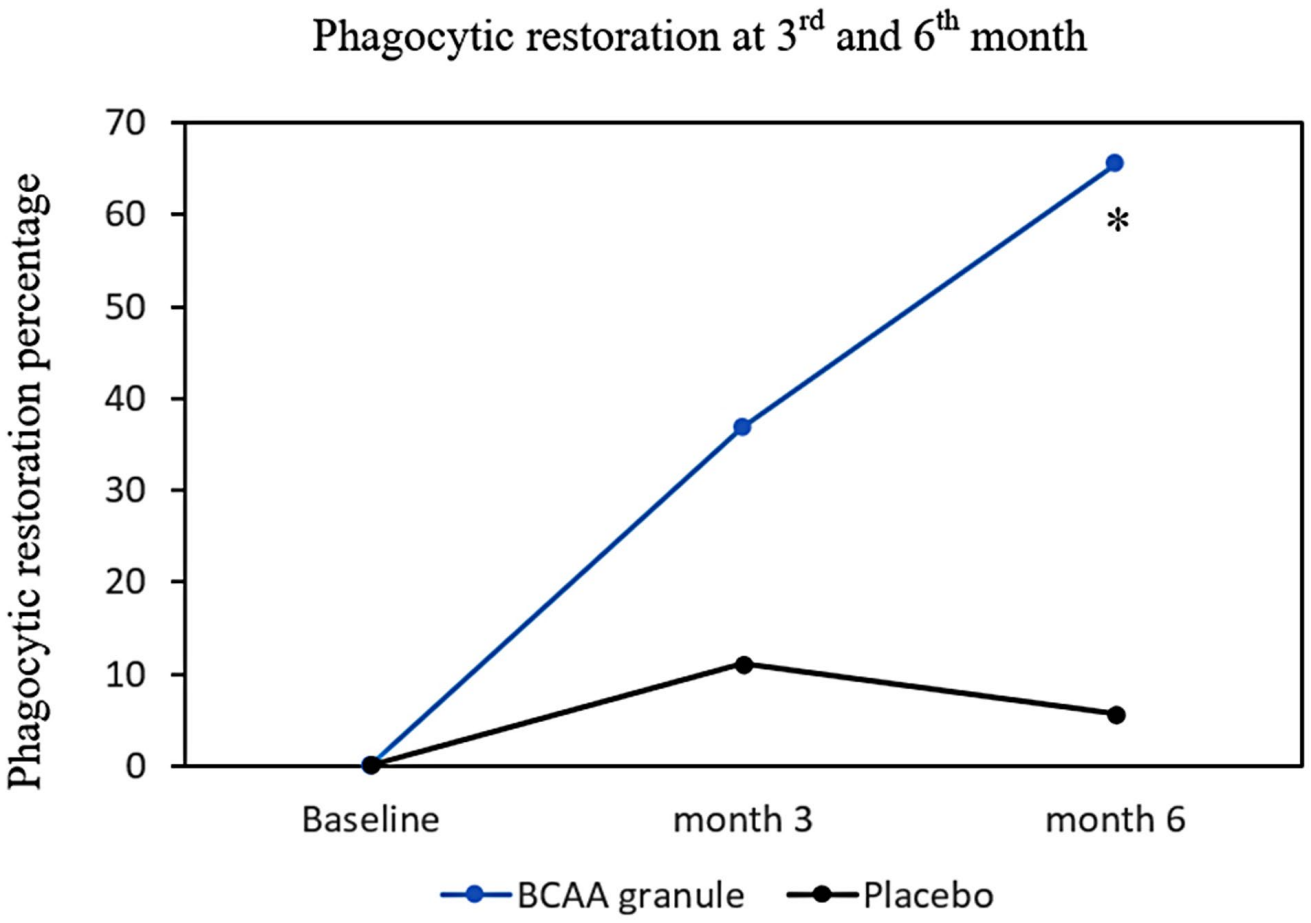
Phagocytic restoration percentage at months 3 and 6. The proportions of phagocytic restoration in both groups at months 3 and 6 were demonstrated in the intention-to-treat analysis. There was a numerical higher phagocytic restoration in BCAA granule group at month 3 (36.8 vs. 11.1%, *p* = 0.124), and statistically higher restoration rate in BCAA granule group than placebo at month 6 (68.4 vs. 5.6%, *p < 0.001). BCAA, branched-chain amino acid.

As a result of phagocytic improvement, the proportion of patients who exhibited phagocytic restoration in the BCAA granule group tended to be higher than that in the placebo group at month 3 (36.8 vs. 11.1%, *p* = 0.124); this proportion increased, and the difference between groups attained statistical significance at month 6 (68.4 vs. 5.6%, *p* < 0.001; Figure 4).

**Figure 5:**
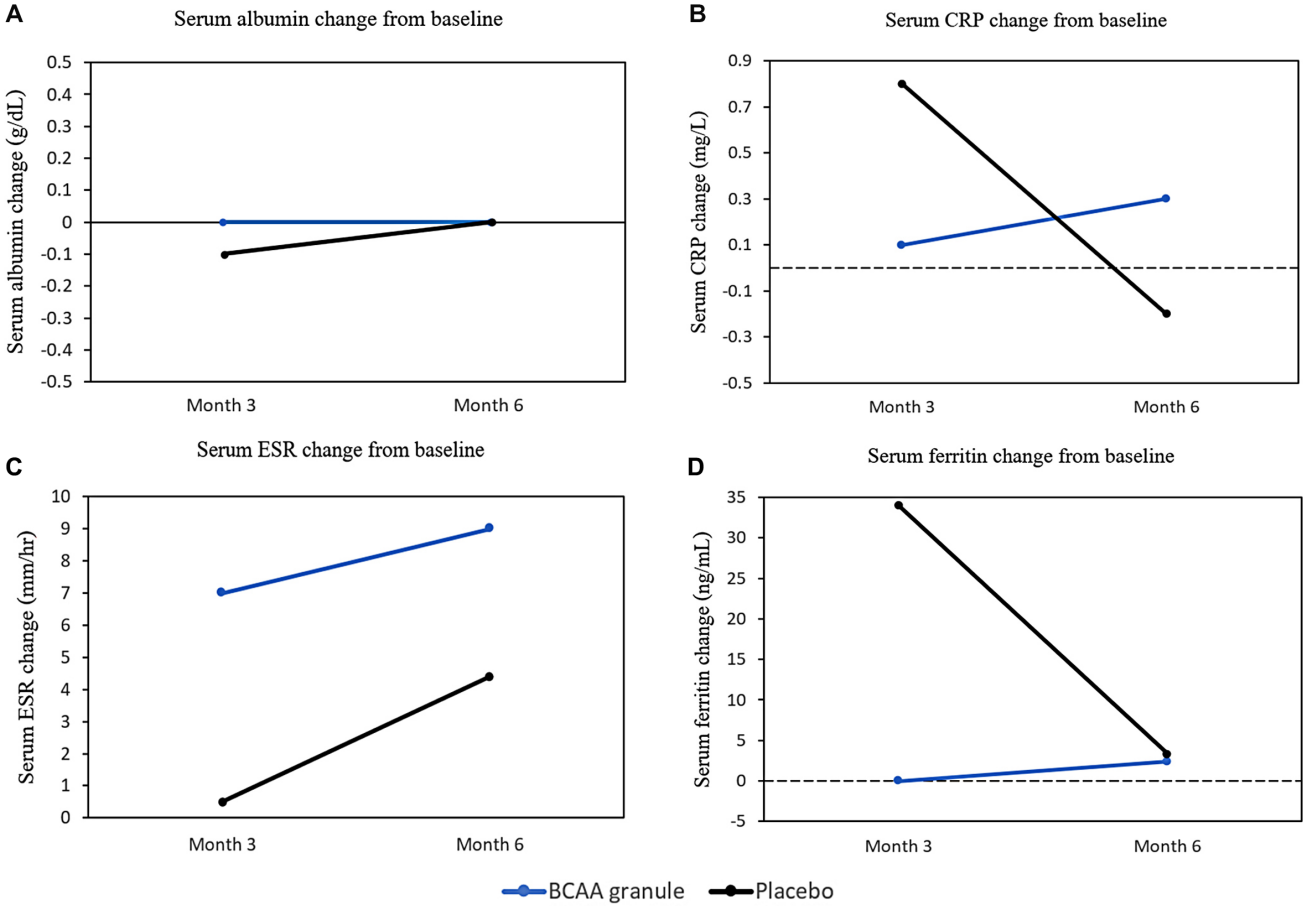
Biochemistry profile changes from baseline at months 3 and 6. **(A)** Changes in serum albumin from baseline at months 3 and 6 in the BCAA granules and placebo groups can be expressed as follows: 0 vs. −0.1 (*p* = 0.807) and 0 vs. 0 (*p* = 0.7), respectively; **(B)** changes in CRP from baseline in months 3 and 6 for BCAA granules and placebo were 0.1 vs. 0.8 (*p* = 0.523) and 0.3 vs. −0.2 (*p* = 0.23), respectively; **(C)** Changes in ESR from baseline in months 3 and 6 for BCAA granules and placebo were as follows: 7 vs. 0.5 (*p* = 0.028) and 9 vs. 4.5 (*p* = 0.254); **(D)** Changes in ferritin from baseline in month 3 and 6 for BCAA granules and placebo were as follows: 0 vs. 34 (*p* = 0.059) and 2.4 vs. 3.4 (*p* = 1), respectively. BCAA, branched-chain amino acids; CRP, C-reactive protein; and ESR, erythrocyte sedimentation rate.

In addition to the analysis of phagocytic activity, comparisons of the validated prognostic score, liver biochemistry, and inflammatory markers were performed. At months 3 and 6, there were no significant differences in AST, ALT, WBC, ANC, MELD-Na, and serum albumin levels between the two groups (Table 2; Figure 5A). The results were in accordance with those pertaining to the inflammatory markers. No clinically relevant changes were observed in ESR, CRP, and ferritin in both groups (Table 2, Figures 5B–D).

**Table 2:** Biochemistry analysis, prognostic score, and inflammatory markers at months 3 and 6.

Variables	BCAA granule group (*n* = 19)	Placebo group (*n* = 18)	*p* value
Month 3			
AST (U/L), mean (SD)	51 (20)	52.7 (20)	0.803
ALT (U/L), median (IQR)	30 (26–41)	32.5 (28–43)	0.41
WBC (x10^3^/μL), mean (SD)	5.1 (1.9)	5.5 (2)	0.55
ANC (x10^3^/μL), mean (SD)	3 (1.2)	3.4 (1.4)	0.382
MELD-Na, mean (SD)	13.4 (4.3)	12.6 (3.7)	0.547
Albumin (g/dL), mean (SD)	3.4 (0.7)	3.6 (0.8)	0.497
ESR (mm/h), mean (SD)	56.6 (42.2)	40.4 (28.6)	0.183
CRP (mg/L), median (IQR)	1.7 (1–4.1)	4.5 (1.5–6.2)	0.287
Ferritin (ng/mL), median (IQR)	219 (92.2–458.5)	225 (128.5–345.2)	0.964
Month 6			
AST (U/L), median (IQR)	43 (37–58)	44 (38–61)	0.975
ALT (U/L), median (IQR)	29.5 (23–36)	29 (23–42)	0.837
WBC (x10^3^/μL), mean (SD)	4.6 (1.6)	5 (2)	0.488
ANC (x10^3^/μL), mean (SD)	2.6 (1)	2.9 (1.7)	0.434
MELD-Na, mean (SD)	13.53 (4.1)	13.83 (5.6)	0.850
Albumin (g/dL), mean (SD)	3.4 (0.7)	3.7 (0.6)	0.312
ESR (mm/h), mean (SD)	58.9 (42.8)	44.5 (31.7)	0.254
CRP (mg/L), median (IQR)	2.3 (0.8–3.3)	2.2 (1–4.1)	0.964
Ferritin (ng/mL), median (IQR)	224 (91.8–460.5)	191 (112–251.8)	0.671

AST, aspartate aminotransferase; ALT alanine aminotransferase; WBC, white blood cell count; ANC, absolute neutrophil count; BCAA, branched chain amino acid; IQR, interquartile range; MELD-Na, Model for End-stage Liver Disease–Sodium score; ESR, erythrocyte sedimentation rate; and CRP, C-reactive protein.

Five hospitalizations due to infection were reported during the study period. Two infectious events were reported in the BCAA granule group, including posttraumatic cellulitis and spontaneous bacterial peritonitis (SBP), both of which occurred within the first 3 months. Three infectious events were reported in the placebo group, including *Vibrio vulnificus* cellulitis with septicemia, SBP, and septic arthritis. Although the number of infectious events was slightly higher in the placebo group than in the BCAA granule group, the difference was not statistically significant (*p* = 0.487). One patient in each group (5%) experienced post-infectious liver decompensation and subsequently died.

Regarding safety profiles, six treatment-emergent adverse events (TEAEs) were reported in 37 patients (16%). No significant difference in adverse events was observed between the BCAA granule and placebo groups (15.8 vs. 16.7%). All reported TEAEs were instances of abdominal distension and bloating; they were all mild and were successfully treated with on-demand prokinetics.

## Discussion

We demonstrated that supplementation with BCAA granules could significantly improve innate immunity in terms of phagocytic activity in patients with cirrhosis. The phagocytic activity among those who received BCAA granules was considerably higher than that among those who received a placebo at 6 months (75.4 vs. 63.4%, *p* < 0.001). Notably, more than two-thirds of patients in the BCAA granule group exhibited phagocytic restoration, while only 5.6% of those in the placebo group achieved this endpoint.

Cirrhosis-associated immune dysfunction is a deleterious cirrhotic complication that increases the susceptibility to infection and further decompensation, leading to significant morbidity and mortality. Several studies have demonstrated that a reduction in phagocytosis may predict the development of infection, organ dysfunction, and 90-day mortality (10). This emphasizes the importance of CAID modulation. Several pathophysiological targets have been proposed to modulate CAID, such as gut bacterial translocation, circulating humoral factors, the bone marrow, immunometabolism, and immune cell signaling (13). However, there are few publications aimed at these emerging immunotherapeutic approaches; this is particularly relevant as several BCAAs have been reported to be considerably beneficial in patients with cirrhosis and exhibit acceptable safety profiles.

This study (The efficacy of Branched-chain amino acid granules to Restore Innate immunity in Cirrhosis patients) is the first randomized controlled study to evaluate the efficacy of BCCA granules as potential novel therapeutic agents for cirrhosis patients with CAID. Our study demonstrated progressive improvement of phagocytic activity in the BCAA granule group from baseline to months 3 and 6. In contrast, there was no significant improvement in phagocytic activity in the placebo group. This result confirms that BCAA granules are efficacious and that this efficacy is time-dependent. These findings are also in line with the results of a previous study (18). Moreover, compared to previous studies, in this study, a significantly higher proportion of patients achieved phagocytic restoration in the BCAA granule group compared to the placebo group at the end of the study (68.4 vs. 5.6%, *p* < 0.001). These results support the use of BCAA granules as potential therapeutic agents to modulate CAID.

Several mechanisms have been reported to underlie the interactions between BCAAs and innate immune functions. These proposed mechanisms may explain the significant efficacy of BCAA granules observed in our study. Firstly, immune cells incorporate BCAAs during the cell proliferation process. Secondly, BCAAs, particularly leucine, can activate the mTOR signal transduction system. Several studies have demonstrated an upstream interplay between mTOR and the immune system (22). It appears that mTOR is a pivotal core protein responsible for integrating diverse microenvironment inputs to support immunity (17). Thirdly, BCAAs can reduce bacterial translocation through intestinal tight junction fortification in rats with cirrhosis (16). And lastly, it has been reported that in rats with cirrhosis, BCAAs can reduce the protein expression of LBP and TLR4, which are the driving factors leading to immune cell exhaustion. These results suggest that BCAAs have multimodal interactions with immune cells. This evidence exemplifies the phagocytic response to BCAA granule supplementation.

Our study also investigated serum albumin levels, MELD-Na, and inflammatory markers. Albumin is an essential component of the metabolic environment of innate immune cells and binds to immunosuppressive mediators such as prostaglandin E2 (PGE2), regardless of its level in the serum (24–26). Therefore, the improvement in phagocytic activity despite the unchanged serum albumin level in our study and a recent study is not unexpected (20). Furthermore, there were no differences in MELD-Na and inflammatory markers between the BCAA granule group and the placebo group, indicating that supplementation with BCAA granules can improve phagocytic activity despite the absence of improvement in the MELD-Na score, serum albumin level, and inflammatory markers.

Our analysis of the clinical outcomes showed that the number of hospitalizations due to infection was not different between the two groups, although there was a significant phagocytic restoration in the BCAA granule group. This finding might be due to several reasons; the two infectious events in the BCAA granule group occurred early during the first 3 months of the study, and the effect of BCAA supplementation on phagocytic function may not yet have ensued. Additionally, most of the participants in our study were in the CTP-A category; such patients seldom experience infectious events, especially in the timeframe of the short follow-up period. Moreover, the sample size in our study was calculated based on phagocytic improvement not the reduction in infectious events. Safety profiles were also assessed. Sixteen percent of the participants reported adverse events, with no significant difference between the two groups.

Our study has some limitations. As the enrolled patients were predominantly diagnosed with CTP-A cirrhosis, a spontaneous phagocytic improvement could have been encountered. In addition, CTP-A cirrhosis patients are at a lower risk of infectious complications than those with decompensated cirrhosis. Therefore, it is difficult to demonstrate a significant reduction in infectious events despite improving phagocytic function, as observed in the current study. The follow-up period in this study was short, so it was difficult to demonstrate a benefit in infectious event outcomes. Therefore, further studies in decompensated cirrhosis with a more extended follow-up period and larger sample size are warranted to determine whether the beneficial effect of BCAA supplementation observed in the current study can be translated into better clinical outcomes in cirrhotic patients.

In summary, our study demonstrated that BCAA granules significantly improved and restored innate immunity in terms of phagocytic activity across various cirrhosis stages, with favorable safety profiles. Further studies should be conducted on clinical outcomes such as infectious events, organ failure, and mortality.

## Data availability statement

The raw data supporting the conclusions of this article will be made available by the authors, without undue reservation.

## Ethics statement

The studies involving human participants were reviewed and approved by Institutional Review Board of the Faculty of Medicine, Prince of Songkla University (REC 62-275-14-1). The patients/participants provided their written informed consent to participate in this study.

## Author contributions

NC, NR, and TP: conceptualization (lead), data curation (lead), formal analysis (lead), methodology (lead), investigation (lead), and writing—original draft (lead). PS: formal analysis (lead) and writing—review (supporting). AK: writing—review (supporting). All authors contributed to the article and approved the submitted version.

## Funding

This study was funded by the Gastroenterological Association of Thailand (GAT), Thai Association for the Study of the Liver (THASL) Research Development Fund, and Faculty of Medicine, Prince of Songkla University, Songkhla, Thailand. The investigational product (administered to the BCAA granule group) was provided by EA Pharma Co., Ltd. EA Pharma Co., Ltd. had no influence on the study design, methodology, data analysis, data interpretation, or manuscript writing in the present study.

## Conflict of interest

The authors declare that the research was conducted in the absence of any commercial or financial relationships that could be construed as a potential conflict of interest.

## Publisher’s note

All claims expressed in this article are solely those of the authors and do not necessarily represent those of their affiliated organizations, or those of the publisher, the editors and the reviewers. Any product that may be evaluated in this article, or claim that may be made by its manufacturer, is not guaranteed or endorsed by the publisher.
